# Supplementary material for: Evaluation of γ-oryzanol Accumulation and Lipid Metabolism in the Body of Mice Following Long-Term Administration of γ-oryzanol
Source: Nutrients. 2019 Jan 6;11(1):104. doi: 10.3390/nu11010104 (PMC6356301; doi:10.3390/nu11010104)
Supplement: Supplementary file 1 [file nutrients-11-00104-s001.pdf]

nutrients

Supplementary Table 1

Analytical conditions for HPLC-MS/MS analysis.

γ-Oryzanol	Cycloartenyl ferulate	24-Methylenecycloartanyl ferulate and its isomers	Campesteryl ferulate	β-Sitosteryl ferulate	Ferulic acid
Precursor ion (m/z) $[M - H]^{-}$	601.5	615.5	575.5	589.5	192.9
Product ion (m/z)	586.3	600.3	560.3	574.3	133.8
Source	ESI	ESI	ESI	ESI	ESI
Ion polarity	Negative	Negative	Negative	Negative	Negative
Declustering potential (V)	-145.0	-145.0	-175.0	-155.0	-70.0
Entrance potential (V)	-10.0	-10.0	-10.0	-10.0	-10.0
Collision energy (V)	-56.0	-58.0	-50.0	-52.0	-22.0
Collision cell exit potential (V)	-27.0	-29.0	-23.0	-31.0	-4.0
Curtain gas (psi)	20.0	20.0	20.0	20.0	30.0
Collision gas (psi)	4	4	4	4	6
Ion spray voltage (V)	-4500.0	-4500.0	-4500.0	-4500.0	-4500.0
Temperature (°C)	600.0	600.0	600.0	600.0	600.0
Ion source gas 1 (psi)	50.0	50.0	50.0	50.0	80.0
Ion source gas 2 (psi)	60.0	60.0	60.0	60.0	70.0

ESI: electrospray ionization.
